# Outdoor and synthetic performance data for PV devices concerning the weather conditions and capacitor values of I-V tracer

**DOI:** 10.1016/j.dib.2023.109007

**Published:** 2023-02-23

**Authors:** C.D. Londoño, J.B. Cano, F. Jaramillo, J.A. Valencia, E. Velilla

**Affiliations:** a Grupo en Manejo Eficiente de la Energía, GIMEL, Universidad de Antioquia UdeA, Calle 70 No. 52-21, Medellín, Colombia; b Centro de Investigación, Innovación y Desarrollo de Materiales – CIDEMAT, Universidad de Antioquia UdeA, Calle 70 No. 52-21, Medellín, Colombia

**Keywords:** Outdoor tests, Photovoltaic devices performance, I-V curves, Capacitive I-V tracers, Weather conditions

## Abstract

This article presents the outdoor and synthetic performance data concerning the main electrical parameters estimated from the I-V curve for three photovoltaic technologies (HIT, m-Si and CIGS) and the weather conditions (irradiance, ambient and panel temperature). Synthetic data were generated by simulating in OpenModelica software the impact of weather conditions on device performance, considering an irradiance range between 50 and 1300 W/m^2^. The outdoor data corresponds to the performance of the evaluated PV modules in outdoor tests in Medellin-Colombia for ten months using capacitive I-V tracers. In both cases, different capacitor values were considered to evaluate the effect on the I-V curve behavior of devices.

**Specifications Table**

**Table:** 

Subject	Renewable Energy, Sustainability and the Environment

Specific subject area	Solar energy, characterization of photovoltaic devices in outdoor and simulating conditions through I-V curves estimated by capacitive technique using different capacitor values
Type of data	Table OpenModelica photovoltaic panel model Python script
How the data were acquired	• The real data were obtained by measuring the electrical performance (I-V curves) for different PV modules in outdoor conditions and eather conditions. Capacitive I-V tracers (details on tracer specifications can be found in [1] and [2]) were used to record the I-V curve of each solar panel. From the curves, the electrical variables concerning the device performance were estimated: short circuit current (*I_sc_*), open circuit voltage (*V_oc_*), maximum power (*P_max_*) and voltage and current at maximum power point (*V_mpp_, I_mpp_*). Ambient and solar panel temperatures were recorded by using PT1000 thermistors (TRITEC International). Solar irradiance was measured by using calibrated cells (Spektron 210 - TRITEC International) coplanar with the solar panel array. Also, a pyranometer class B was used to validate the irradiance values. All variables were sampled at a rate of 1 sample/minute during the light-hours (6:00AM to 6:00PM). • The synthetic data were obtained by simulating the solar panels behavior in OpenModelica software through python scripting. In both cases (outdoor and synthetic data) different values of capacitance were used for the I-V tracer.
Data format	Raw
Description of data collection	Data correspond to the electrical variables of three modules of different technologies, Panasonic VBHN330SJ47 (HIT), Znshine solar ZX55(17.8)M (m-Si), and Miasolé FLEX–02 120 N (CIGS) in outdoor and simulating conditions. For outdoor data, the panels were located in natural sunlight without a tracker, facing south at a fixed tilt angle of 13°. Different capacitance values were used in outdoor tests and simulation process to estimate the device performance.
Data source location	Institution: Universidad de Antioquia (UdeA) City/Town/Region: Medellin Country: Colombia Latitude and longitude for collected samples/data: **6°15′ 38″N 75°34′ 05″W**
Data accessibility	Data is hosted on Mendeley Data [3]. Data identification number: 10.17632/k8y3nxxyp5.2 Direct URL to data: https://data.mendeley.com/datasets/k8y3nxxyp5/2 The source code for generating the simulated data is include in the link above.
Related research article	Londoño, C. D., Cano, J. B., Velilla, E., Capacitive tracer design to mitigate incomplete IV curves in outdoor tests. Sol. Energy. *243* (2022), 361–369. 10.1016/j.solener.2022.08.021

## Value of the Data

• The dataset contains the electrical performance estimated from the I-V curve for three different commercial photovoltaic technologies (HIT, m-Si, CIGS) concerning the weather conditions. Synthetic data were generated in an electrical simulation program (OpenModelica). The data are highly valuable to research, monitor and compare the behavior of these technologies and devices under the same conditions.
• These data can benefit researchers and companies working on the characterization and validation of photovoltaic technologies in outdoor conditions as well as other applications related to Deep Learning and solar panel modeling.
• These data can be used to reproduce previous results in order to validate the proposed design approach for the capacitive I-V tracers [4]. In addition, because the data correspond to the outdoor performance of PV devices, the data can be used for comparing the PV devices’ behavior under real operation conditions or selecting proper conditions regarding the weather conditions.

## Objective

Incomplete I-V curves of PV devices were observed in outdoor tests when capacitive tracers were used to trace the I-V curve. This behavior was also observed by simulating the PV devices considering similar weather conditions and the circuital elements involved in the capacitive technique. In this regard, the synthetic data allows validating the outdoor behavior and the proposed methodology for sizing the capacitance of the tracer [4]. Therefore, both datasets are useful for researchers to understand, compare and model the performance of devices under real weather conditions. In addition, datasets can be used for validating testing techniques to be implemented in I-V tracers.

## 1 Data Description

**Fig. 1:**
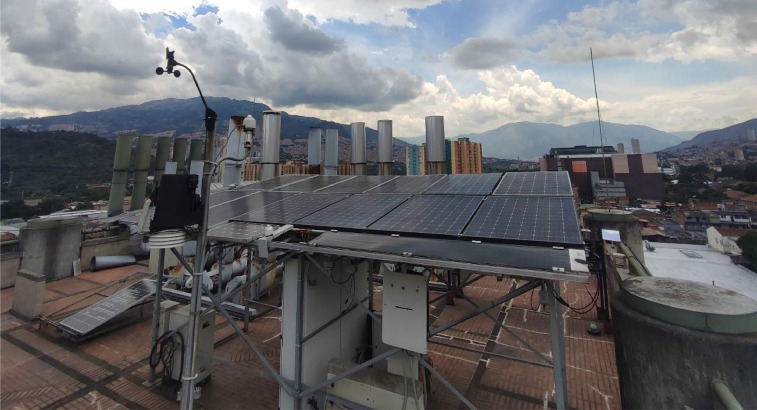
Laboratory for performance characterization of photovoltaic devices in outdoor conditions, Sede de Investigación Universitaria – SIU, Universidad de Antioquia, Medellín, Colombia.

The file “*BD_DIB_Outdoor_Solar_Panels_Electrical_Parameters.xlsx”* contained in the Mendeley Data repository [3], it is related to the outdoor dataset with the electrical parameters of three different photovoltaic panels (Panasonic VBHN330SJ47 (HIT), Znshine solar ZX55(17.8)M (m-Si), and Miasolé FLEX–02 120 N (CIGS)) and weather conditions related to the place where these devices were installed (Fig. 1). In Table 1 are listed the details relating to the data. This table comprises the column names as they appear in the data CSV file, the unit of measurement, and a brief description of the data in each column.

**Table 1:** List of column names, units of measurement and descriptions of data provided in the outdoor dataset (*“BD_DIB_Outdoor_Solar_Panels_Electrical_Parameters.xlsx”*).

Column Name	Units	Description
Panel	–	Technology / panel device
C	µF	Capacitance used in the I-V tracer
datetime	yyyy-mm-dd HH:MM:SS	Measurement date and time
Irradiance	W/m^2^	Global irradiance coplanar to tilt angle of the devices
T panel	°C	Panel temperature
T amb	°C	Ambient temperature
Voc	V	Open circuit voltage
Isc	A	Short circuit current
Pmax	W	Maximum power
Vmpp	V	Voltage at the maximum power point
Impp	A	Current at the maximum power point
Vmin	V	Minimum voltage measured in I-V curve
Imin	A	Minimum current measured in I-V curve
Voc_Success	%	Success rate of voltage, defined by $100(1-\frac{I_{min}}{I_{sc}})$
Isc_Success	%	Success rate of current, defined by $100(1-\frac{V_{min}}{V_{oc}})$

The file “*BD_DIB_Synthetic_Solar_Panels_Electrical_Parameters.xlsx”* contained in the Mendeley Data repository [3], it is related to the synthetic dataset with the electrical parameters for three different photovoltaic panels (Panasonic VBHN330SJ47 (HIT), Znshine solar ZX55(17.8)M (m-Si), and Miasolé FLEX–02 120 N (CIGS)) estimated in OpenModelica [5] software version 1.19.2. In Table 2 are listed the details relating to the data. This table comprises the column names as they appear in the data CSV file, the unit of measurement, and a brief description of the data in each column.

**Table 2:** List of column names, units of measurement and descriptions of data provided in the simulating dataset (*“BD_DIB_Synthetic_Solar_Panels_Electrical_Parameters.xlsx”*).

Column Name	Units	Description
Panel	–	Technology / panel device
C	µF	Capacitance used in the I-V tracer
Irradiance	W/m^2^	Global irradiance coplanar to tilt angle of the devices
Voc	V	Open circuit voltage
Isc	A	Short circuit current
Pmax	W	Maximum power
Vmpp	V	Voltage at the maximum power point
Impp	A	Current at the maximum power point
Vmin	V	Minimum voltage measured in I-V curve
Imin	A	Minimum current measured in I-V curve
Voc_Success	%	Success rate of voltage, defined by $100(1-\frac{I_{min}}{I_{sc}})$
Isc_Success	%	Success rate of current, defined by $100(1-\frac{V_{min}}{V_{oc}})$
Tmpp	ms	Time to reach the maximum power point
TVoc	ms	Time to reach the maximum open circuit point

The file *“IVTraceCapacitor.mo”* contained in the Mendeley Data repository [3], it is the OpenModelica [5] photovoltaic panel model that allows to model the behavior of the photovoltaic modules. The parameters of modules at Standard Test Conditions are show in Table 3.

**Table 3:** Parameters of photovoltaic modules.

Panel Technology	Manufacturer	Series	Electrical Parameters at Standard Test Conditions (STC)					Dimensions (mm)	
			P_max_ (W)	V_mpp_ (V)	I_mpp_ (A)	V_oc_ (V)	I_sc_ (A)	Length	Width
CIGS	Miasolé	FLEX–02 120N	120	30.50	3.93	38.10	4.53	2598	370
m-Si	Znshine solar	ZX55(17.8)M	55	17.80	3.09	22.25	3.34	837	541
HIT	Panasonic	VBHN330SJ47	330	58.00	5.70	69.70	6.07	1590	1053

**Fig. 2:**
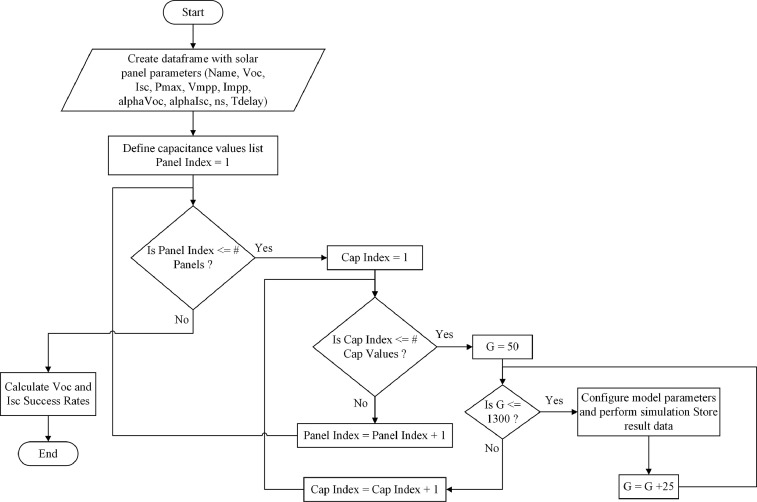
Flowchart of simulating process in OpenModelica [5] using the python script *“Generate_Synthetic_Data_OpenModelica.py”*.

The file *“Generate_Synthetic_Data_OpenModelica.py”* contained in the Mendeley Data repository [3], it is a python script related to the simulation process carried out to estimate the synthetic dataset data. This script is summarized in the flowchart shown in the Fig. 2.

**Fig. 3:**
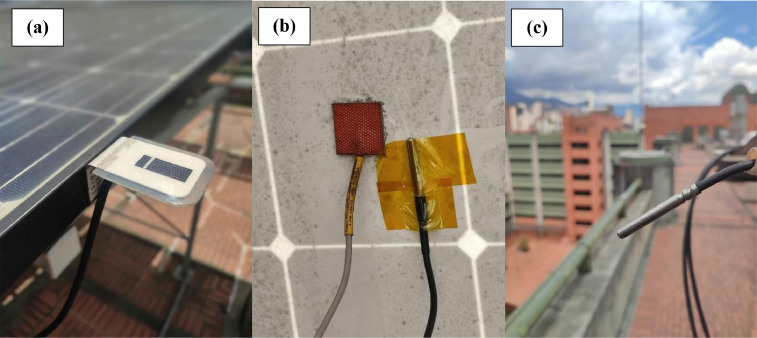
Sensors for measuring weather conditions. Panel (a) corresponds to irradiance sensor, panel (b) to panel temperature sensor and panel (c) to ambient temperature sensor.

**Fig. 4:**
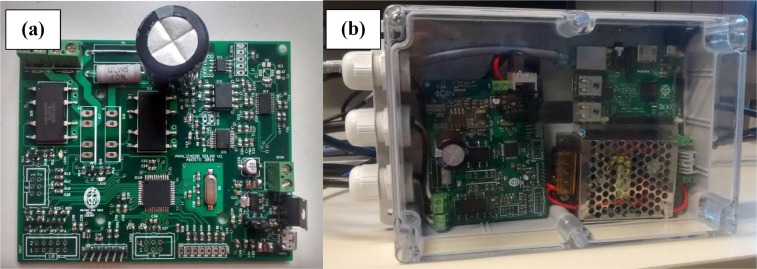
Capacitive I-V tracer. Panel (a) corresponds to the designed electronic circuit to trace the I-V curve considering the capacitive technique. Panel (b) shows the developed prototype to record the I-V curves in outdoor conditions using an embedded computer to automate the process.

**Fig. 5:**
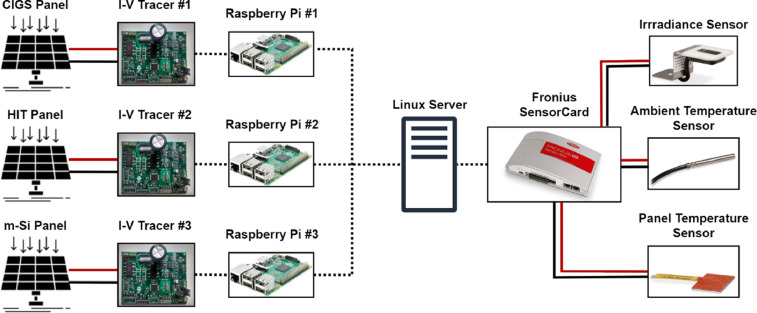
Schematic diagram of the experimental setup for measuring real I-V curves in outdoor conditions and weather variables.

**Fig. 6:**
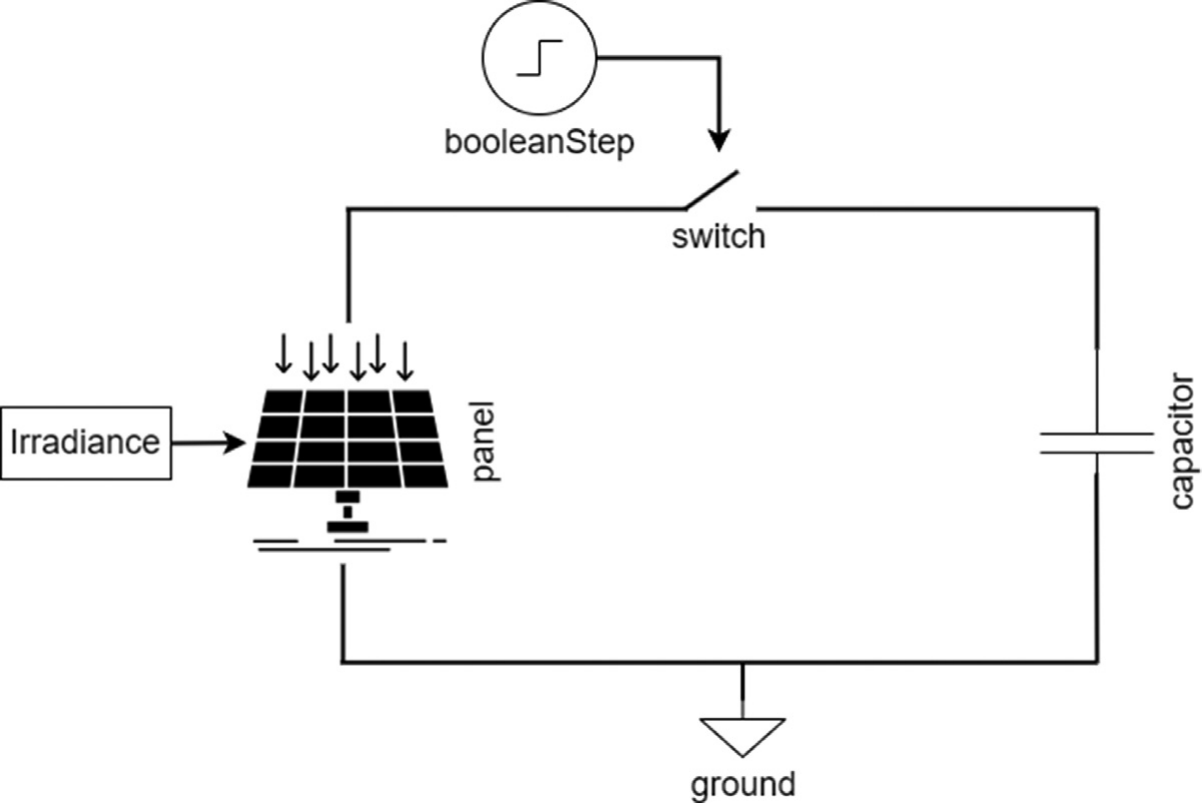
Circuit diagram used in OpenModelica by python scripting.

The experimental setup used to obtain the data is described in the next section. In this regard, Fig. 3 shows the weather condition sensors, Fig. 4 shows the developed I-V curve tracers and Fig. 5 shows the implemented monitoring system. Concerning the synthetic data, Table 4 shows the main parameters considered in OpenModelica to perform the simulations following the flowchart shown in Fig. 2 and the circuit diagram of Fig. 6.

**Table 4:** Main parameters for simulating processes in OpenModelica.

Parameter	Values
Irradiance Range	50–1300 W/m^2^ with steps of 25 W/m^2^
Capacitances	1500 µF, 3300 µF, 4400 µF, 4700 µF, 6600 µF, 6800 µF, 8200 µF, 13,600 µF, 14,800 µF
Solar Panels Parameters	CIGS - Miasolé FLEX–02 120 N I_sc_ = 4.53A, V_oc_ = 38.1 V, I_mpp_ = 3.93A, V_mpp_ = 30.5 V, alphaVoc = −0.0028, alphaIsc = 0.00008, ns = 60
	m-Si - Znshine solar ZX55(17.8)M I_sc_ = 3.34A, V_oc_ = 22.25 V, I_mpp_ = 3.09A, V_mpp_ = 17.8 V, alphaVoc = −0.0028, alphaIsc = 0.00008, ns = 24
	HIT - Panasonic VBHN330SJ47 I_sc_ = 6.07A, V_oc_ = 69.7 V, I_mpp_ = 5.7A, V_mpp_ = 58 V, alphaVoc = −0.0028, alphaIsc = 0.00008, ns = 96
Sampling	Up to 1000 samples at a sampling rate of 0.322 ms/sample
*t_delay_*	1.5 ms (except for HIT with 5 ms). According to typical values of solid-state relay in the datasheet.

## 2 Experimental Design, Materials and Methods

This section presents the methodologies, methods and elements used for the information acquisition process of the real data in outdoor conditions and the data obtained through simulation.

### 2.1 Outdoor Data

The outdoor data has been obtained using the facilities of the Universidad de Antioquia for photovoltaic outdoor device characterization, located in Medellín, Colombia (6°15′ 38″N 75°34′ 05″W). Fig. 1 shows the entire experimental set-up for monitoring the PV devices, where it can be observed photovoltaic devices under test, sensors for measuring weather variables and electronic power devices for connecting panels to the grid.

Fig. 3 shows the sensor used for measuring temperature and irradiance. Panel and ambient temperature were measured by PT1000 thermistors (TRITEC International). In the panel temperature case, the thermistor was located in the back side of the panel. Global irradiance was measured by using a calibrated cell (Spektron 210 - TRITEC International) located parallel to the tilt angle of the panels. The three sensors of Fig. 3 were connected to a Fronius SensorCard (Fronius International GmbH). These variables were measured every minute and were synchronized regarding the I-V tracers records.

### 2.2 Photovoltaic Modules

Table 3 shows the characteristics of the three photovoltaic modules used in the development of this work.

### 2.3 I-V Tracers

Fig. 4 shows the equipment used to measure the I-V curves of the solar panels that were evaluated. The I-V tracers (Fig. 4a) use the capacitive technique that consists in tracing the I-V curve by measuring the charge of the capacitor connected to the device under test. For more details about the characteristics of these device consult [1,2]

The capacitance values installed in the I-V tracers during the exposure time for each evaluated solar panel were as follows: for Miasolé FLEX–02 120 N (CIGS) panel 3300, 4400, 8200 and 14,800 µF, for Znshine solar ZX55(17.8)M (m-Si) panel 1000, 4400, 8200 and 14,800 µF and for Panasonic VBHN330SJ47 (HIT) panel 3200, 4700, 6800 and 13,600 µF. The capacitance values were changed with the aim of validating the capacitance design methodology proposed in [4].

### 2.4 Experimental Setup

Fig. 5 shows a schematic diagram of the experimental setup used in the development of this work. Each panel is connected to an I-V tracer (with its corresponding capacitor) whit the corresponding raspberry pi to record the I-V curve data and send the data to the main computer (linux server). Ambient and panel temperature and irradiance sensors were connected to a Fronius SensorCard to record the weather data. Each I-V curve and weather variables were measured simultaneously every minute during daylight hours (6:00AM to 6:00PM).

### 2.5 Synthetic Data

The synthetic data were generated by using OpenModelica simulation software. Fig. 6 shows the circuit diagram representation of the simulation model. The solar panel, capacitive load and the switch are present in the process. The simulation model contains different blocks that allow modification and set parameters by python scripting (using the OMPython package [6]). The booleanStep block controls the switch, guaranteeing its closure at *t = 0 s*. The irradiance block allows to set a constant irradiance for the simulation, while the ModuleData block allows to modify the main panel characteristics (*V_oc_, I_sc_, V_mpp_, I_mpp_* and temperature coefficients). Solar panel models were taken from the Photovoltaics library described at [7] and parameterized using the datasheet information for each panel.

.The simulation process in OpenModelica was automatized by using a Python script according to the flowchart shown in Fig. 2. By the end of the simulation a dataset with the parameters was generated as shown in the Table 2. The source code and the OpenModelica model file “.om” can be download from the repository listed in the Specifications table/ data accessibility section [3].

Table 4 shows the parameters used for the simulation by python scripting using the OpenModelica library.

## CRediT authorship contribution statement

**C.D. Londoño:** Methodology, Software, Investigation, Writing – original draft. **J.B. Cano:** Conceptualization, Methodology, Software, Investigation, Writing – review & editing, Supervision. **F. Jaramillo:** Investigation, Writing – review & editing, Supervision. **J.A. Valencia:** Validation, Supervision, Writing – review & editing. **E. Velilla:** Conceptualization, Methodology, Software, Validation, Supervision, Writing – review & editing.

## Declaration of Competing Interest

The authors declare that they have no known competing financial interests or personal relationships that could have appeared to influence the work reported in this paper.

## Data Availability

Outdoor and Synthetic Data of PV devices Performance Concerning the Weather Conditions and Capacitor Values of I-V Tracer (Original data) (Mendeley Data).
